# Preoperative Group Consultation Prior to Surgery for Colorectal Cancer—an Explorative Study of a New Patient Education Method

**DOI:** 10.1007/s13187-020-01951-7

**Published:** 2021-01-14

**Authors:** Sofie Walming, Eva Angenete, David Bock, Mattias Block, Hanna de la Croix, Anette Wedin, Eva Haglind

**Affiliations:** 1 Department of Surgery, SSORG - Scandinavian Surgical Outcomes Research Group, Institute of Clinical Sciences, Sahlgrenska Academy, University of Gothenburg, Gothenburg, Sweden; 2 Department of Surgery, Helsingborg Hospital, Helsingborg, Sweden; 3 Department of Surgery, Sahlgrenska University Hospital, Region Västra Götaland, Gothenburg, Sweden

**Keywords:** Rectal neoplasm, Cancer, Oncology, Health education

## Abstract

**Abstract:**

Patients with colorectal cancer may lack information about the disease and treatment. In 2017, a group consultation before start of surgery was introduced at a university hospital in western Sweden to inform about the disease, treatment, and ongoing scientific studies. The primary aim of this study was to explore the experience of the patients attending the group consultation. Based on semi-structured interviews with patients with colorectal cancer, a questionnaire was constructed and administered to patients, both those attending and those not attending the group consultation. In total, 124 patients were included and the response rate was 86%. A majority of patients attending the group consultation would recommend it to someone else with the same illness. Of the patients attending the group consultation, 81% (30/37) patients agreed, fully or partially, that attending the group consultation had increased their sense of control and 89% (33/37) that the information they received at the group consultation increased their feeling of participation in the treatment. Preoperative group consultation is a feasible modality for informing and discussing the upcoming treatment for colorectal cancer with the patients, and the patients who attended the group setting appreciated it. Attending the group consultation increased the patients’ feeling of active participation in their treatment and their sense of control, which could possibly both improve their experience of their illness and facilitate recovery.

**ClinicalTrials.gov identifier:**

NCT03888313

## Introduction

Patients with colorectal cancer receive information at different occasions during the course of the care, both before, during, and after treatment, to increase their ability to cope with their illness and to promote recovery [1]. Group consultation is a comprehensive term to describe care models where several patients are seen by one or more clinicians at the same time. Ideally, the group consultation should deliver support from other patients as well as all other things included in the usual healthcare [2].

Patients with colorectal cancer have been found to lack information on the disease and the treatment [3–5]. Patients with poorer physical status as indicated with a higher ASA score, and patients living without a partner, are less satisfied with the information received [3, 6]. Preoperative information and education as a method of improving outcomes for treatment of colorectal cancer has seldom been studied, but one study of patients receiving a stoma concluded preoperative education was more effective than the traditional education given postoperative [7]. Patients receiving radiotherapy and randomized to a diagnose-specific group consultation as well as individual information compared to standard information, were more satisfied compared to patients who received standard information [8]. In a review of health-related quality of life (HRQoL) in patients surviving cancer, it was reported that studies on information and quality of life did not find any positive association between information provision and *physical* HRQoL [9]. In the same review, however, it was reported that studies have shown that patients receiving clear and high levels of information had better *mental* and *global* HRQoL [9, 10].

The specific background to this study is that in 2017, a group consultation inviting patients planned for elective surgery for colorectal cancer was launched at a University Hospital in western Sweden. The consultants and nurses that participated in the group consultation expressed verbally that they found patients appreciative. However, before deciding whether to include the preoperative group consultation into usual care at the University Hospital, an explorative study of the patients’ experience was deemed important. The aim of this study was to investigate how the patients with recently diagnosed colorectal cancer experienced the preoperative group consultation.

## Methods

The group consultation was held in a conference room at the hospital. A consultant surgeon and a research nurse were present, and the patients were invited to bring along family members. The session began with a presentation including information about the postoperative mobilization and breaking of fast. When needed, the importance of smoke cessation before surgery was also discussed. This was followed by questions from the patients and relatives about things related to colorectal cancer and the planned treatment. After this questions-and-answers part, information about ongoing clinical trials were given, orally as well as the formal written patient information document.

The data from the questionnaires were supplemented by data retrieved from medical records. The study-specific questionnaire consisted of questions previously used [11, 12] and newly generated questions, developed according to an established method [13, 14]. The new questions were developed by in-depth interviews with patients with colon or rectal cancer before they had gone through surgery. The verbatim transcript of the interviews underwent content analysis, and new questions were constructed based on the themes identified. An expert group selected the questions to be included in the questionnaire, which was then subject to face validation. The face-to-face validation ensured that the questions were easy to comprehend and straightforward to answer. An instrument for assessment of quality of life was included, the five dimensions of the EQ-5D-5L as well as the EQ VAS [15], and questions on socioeconomics and comorbidity. After revisions, the final version of the questionnaire was printed and used in the study. The patients who chose not to attend the group consultation but consented to be included in the study received a specific questionnaire, without questions related to the group consultation.

### Patients and Statistics

All patients planned for surgery for colorectal cancer at a university hospital in Sweden were invited to participate in the group consultation on the Friday of the week they had their primary consultation. The patients who chose to attend the group consultation were informed about, and invited to participate in, the study during the group consultation; if they accepted, they also received the questionnaire. The patients who chose not to attend the group consultation were informed and invited to participate in the study at their next visit in the routine pathway (the outpatient pre-operative work-up visit), where they were given the specific questionnaire after informed consent. All questionnaires were accompanied by a pre-paid return envelope. All patients who mailed the questionnaire to the research secretariat received a thank you post card. When possible, patients who had not returned the questionnaire received a reminder either by post card or telephone. No reminder post cards were sent out after the patients undergone surgery.

Since the aim of the study was to explore the experience of the patients attending the group consultation rather than explicit statistical hypothesis testing, no a priori calculation of power was performed. Descriptive statistical methods were used for presenting the demography and the outcome measures, SPSS and R version 3.2.3 [16] were used. In R, the ggplot2 and tidygeocoder packages were used.

The study was preregistered at www.clinicaltrials.gov (NCT03888313). Permission was obtained from the Regional Ethical Review Board in Sweden (2019-00665/1127-18). Permission to use the EQ-5D 5L [15] was obtained.

## Results

**Fig. 1:**
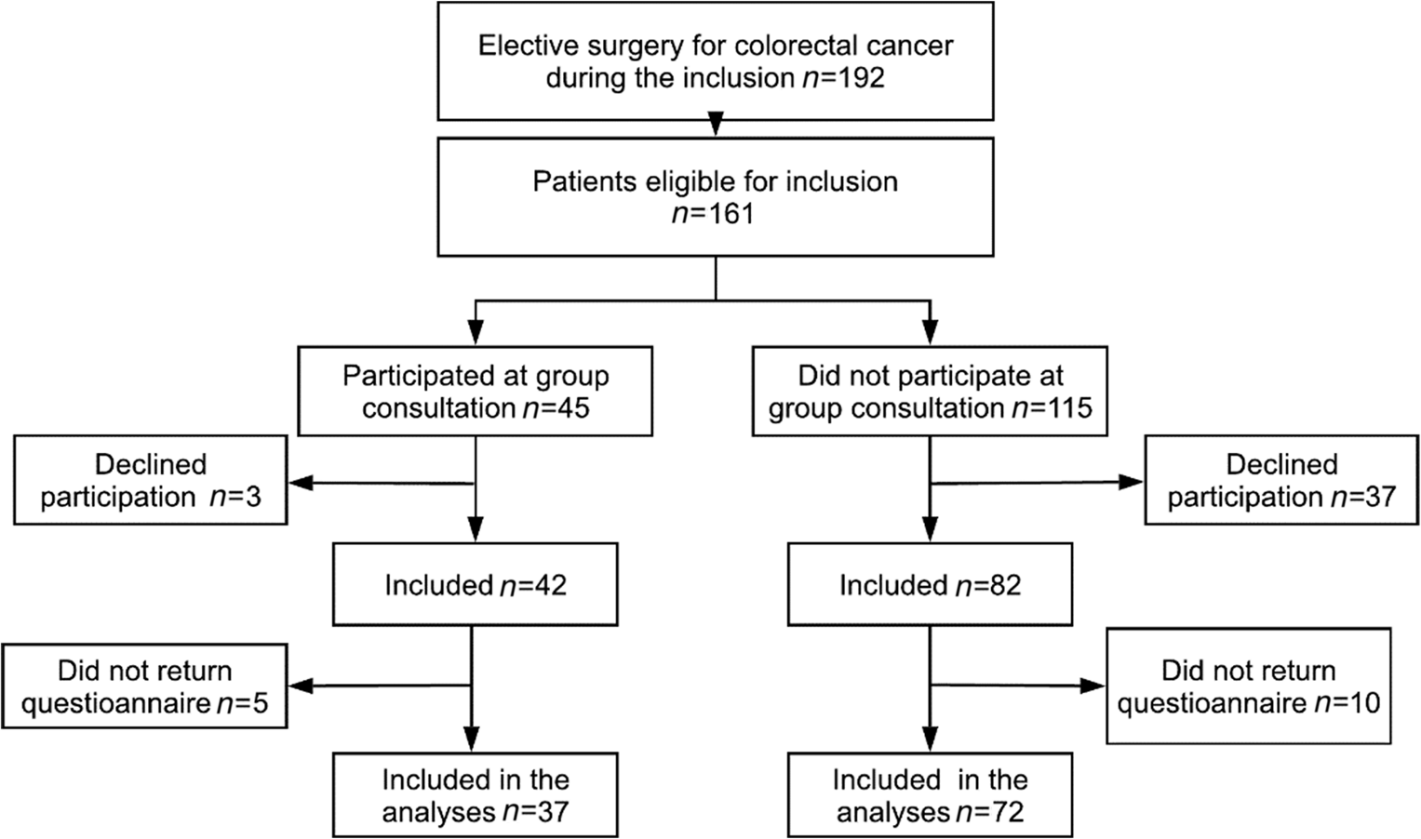
Flow chart of patients. The overall response rate was 88%

In total, 124 patients were included from April to November 2019. Forty-two patients attended the group consultation and 82 did not. Fifteen patients did not return the questionnaire, rendering a response rate of 86% (Fig. 1). In relation to their first visit at the outpatient clinic, the patients not attending the group consultation completed the questionnaire later than the ones who attended the group consultation (11/72 vs 11/37 within 4 days).

**Fig. 2:**
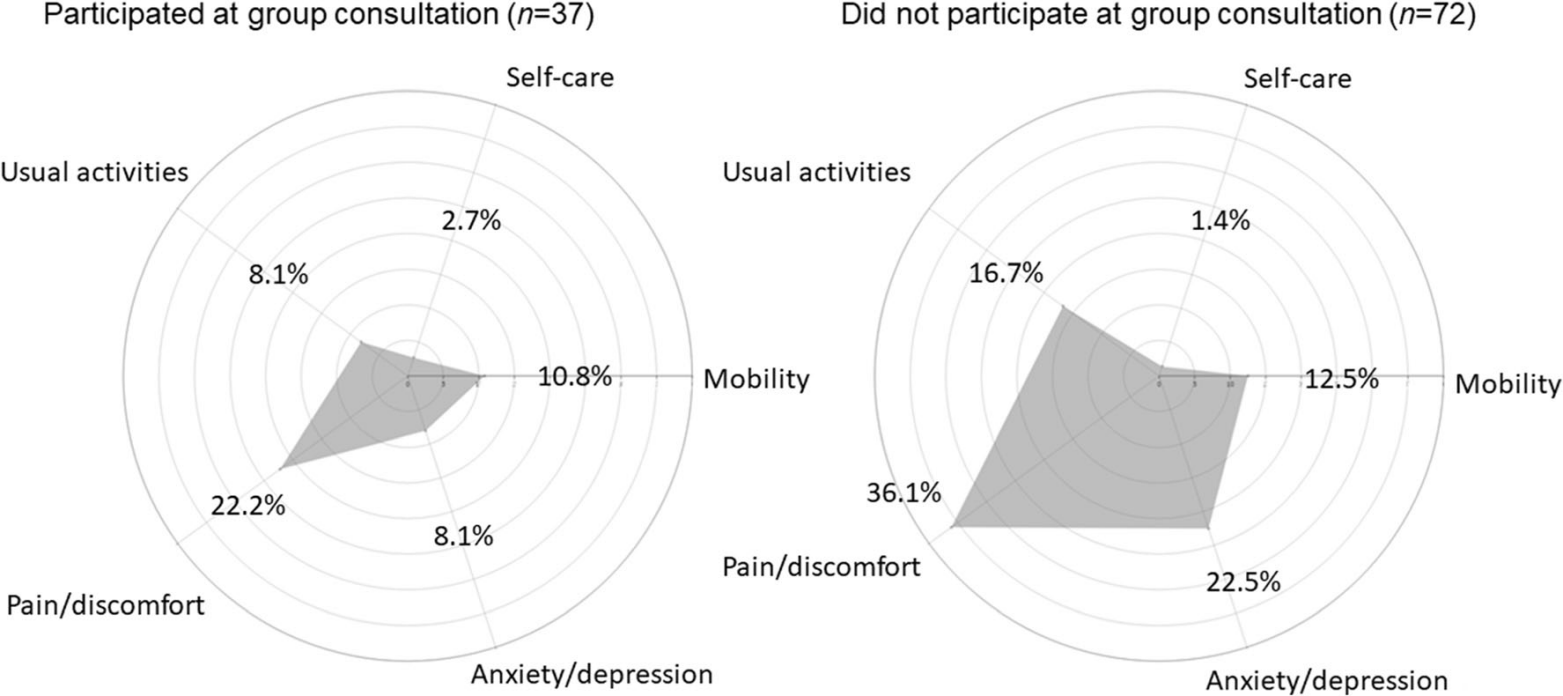
The five dimensions of the EQ-5D 5L. Presented as proportion of patients in the group who stated, “I have moderate problems”, “I have severe problems”, or “I have extreme problems” with the domain

Among the patients who attended the group consultation, there was a tendency that a larger proportion of patients had tertiary or university education (Table 1). The patients attending the group consultation had more comorbidity than the patients who did not attend the group consultation, and their distance to the hospital was shorter. More patients, who did not attend the group consultation, were subject to abdominoperineal resection and had preoperative radiation or chemotherapy. The EQ VAS for the patients who attended the group consultation was median 75.0 (IQR 62.5–85.0, *n* = 37) and for the patients that did not attend the group consultation median 70.0 (IQR 58.8–80.0, *n* = 70). The results of the five dimensions of the EQ-5D 5L are presented in Fig. 2. The results indicate that the group who chose not to attend the group consultation more often experienced pain, anxiety/depression, and difficulties performing activities of daily life.

**Table 1:** Demography and clinical characteristics of included patients (*n* = 124). Data were collected from medical records and the questionnaires distributed before start of treatment

	Attended the group consultation (*n =* 37)	Did not attend the group consultation (*n =* 72)	Drop-outs (*n* = 15)	Missing data
Age, median (range)	68 (41–85)	67 (35–87)	68 (51–82)	0
Male; female	20; 17	38; 34	8; 7	0
Distance (km) to hospital (mean, min–max)	9.9 (5.0–38.3)	34.7 (1.2–412.2)	40.7 (1.2–220.7	0
Smoker	2 (5)	2 (3)	NA	1
Comorbidity*	28 (76)	43 (60)	NA	0
Depression	7 (19)	14 (19)	NA	1
Tertiary or university education	16 (46)	24 (34)	NA	3
Employed	11 (30)	23 (32)	NA	0
*Diagnosis*				0
Colon cancer	19 (51)	37 (51)	1 (7)	
Rectal cancer	17 (46)	35 (49)	14 (93)	
Other	1 (3)	0	0	
Preoperative staging**				
T1-2	11 (33)	15 (24)	3 (20)	14
T3	18 (55)	24 (39)	6 (40)	14
T4	4 (12)	23 (37)	6 (40)	14
N0	25 (69)	43 (65)	8 (53)	7
N1-N2	11 (31)	23 (35)	7 (47)	7
M0	29 (78)	56 (80)	13 (87)	2
MX or M1	8 (22)	14 (20)	2 (13)	2
Preoperative radiotherapy	9 (24)	26 (36)	10 (67)	0
Preoperative chemotherapy	1 (3)	12 (17)	4 (27)	0
*Surgical procedure*				0
Hemicolectomy	8	19	1	
Colectomy	2	1	0	
Resection of sigmoid colon	9	10	1	
Anterior resection	10	17	3	
Hartman’s procedure	2	6	3	
Abdominal perineal resection	5	15	5	
Other	1	4	2	
Postoperative chemotherapy	12 (41)	32 (46)	3 (25)	13

*Such as acute myocardial infarction, pulmonary disease and stroke. *NA*, not applicable due to questionnaire based data. **According to medical records and X-ray reports

Two-thirds of the patients collected further information about cancer and treatment from other sources than healthcare, and this finding was consistent among the two groups (Table 2). None of the patients had been in contact with patient organizations. Results indicate that a larger proportion of patients among the ones that did not attend the group consultation browsed the internet and the webpage provided by the public healthcare called the Care guide (www.1177.se), than those who attended the group consultation.

**Table 2:** Communication with the surgeon and contact nurse at the outpatient clinic and the patients’ sources of information. Data were collected from the questionnaire distributed to patients who attended the group consultation and to patients who did not attend

	Attended the group consultation (*n =* 37)		Did not attend the group consultation (*n =* 72)		Total	
	Yes	No/Not yes or no/Don't know	Yes	No/Not yes or no/Don't know		Missing
Did you receive sufficient information about the planned treatment at your visit to the Surgical clinic?	33 (89)	4	72 (100)	0	109	0
Do you feel that communicating with the doctors at the Surgical clinic has worked well?	36 (97)	1	65 (90)	7	109	0
Do you feel that communicating with the contact nurses at the Surgical clinic has worked well?	32 (86)	5	66 (92)	6	109	0
Have you received sufficient information about how to wash yourself before the operation?	36 (97)	1	70 (97)	1	108	1
Have you received sufficient information about when to start eating and drinking after the operation?	32 (86)	5	60 (83)	12	109	0
Have you received sufficient information about the fact that you are to get up and walk in the corridor of the ward the day after the operation?	36 (97)	1	70 (97)	2	109	0
**Which sources of information have you used to search for knowledge about your illness and treatment?**	**Attended the group consultation (*n*** **= 37)**		**Did not attend the group consultation (*n*** **= 72)**		**Total**	**Missing**
	**Yes**		**Yes**			
Books and newspapers	2 (5)		4 (6)		6 (6)	0
TV programmes	1 (3)		1 (1)		2 (2)	0
Internet searches (e.g., www.google.se)	11 (30)		30 (42)		41 (38)	0
The Care guide (www.1177.se)	6 (16)		24 (33)		30 (28)	0
Wikipedia (www.wikipedia.org)	1 (3)		5 (7)		6 (6)	0
Internet forum (e.g., www.familjeliv.se)	0		1 (1)		1 (1)	0
Patient association (e.g., ILCO)	0		0		0	0
Cancer Society/Foundation	3 (8)		8 (11)		11 (10)	0
Social media (e.g., Facebook)	1 (3)		3 (4)		4 (4)	0
Talked to someone I know who has knowledge	15 (41)		21 (29)		36 (33)	0
Other source of information	4 (11)		3 (4)		7 (6)	0
I have not searched for additional information	12 (32)		25 (35)		37 (34)	0

*Yes = four or fewer days, No = more than four days or Don't know. Values in parentheses are percent

Among the patients attending the group consultation, 15/36 stated that they wanted information both at the group consultation and at their ordinary visit to the outpatient clinic. Further, 2/36 stated that they wanted information on scientific studies at the out-patient clinic only and 9/36 at the group consultation only.

Of the 37 patients attending the group consultation, 35 would recommend someone with the same condition to attend (Table 3). About 90% agreed, fully or partly, that their participation at the group consultation increased their understanding of the planned treatment (34/37) and increased their feeling of involvement in the treatment (33/37). Three patients stated that their participation in the group consultation did not increase their sense of control; however, the remaining 80% (30/37) agreed, fully or partly, with the statement about increased sense of control. A majority (24/37) were positive regarding group consultation; 19/37 also stated that it was possible to raise difficult questions, and 21/37 appreciated meeting others with the same disease.

**Table 3:** Experience of the group consultation. Data were collected from the questionnaires distributed to the patients participating in the group consultation

	Frequencies						
	Totally correct	%	Partially correct	Not correct	Don’t know	Total	Missing
Attending the information clinic improved my understanding about the upcoming treatment	19	51	15	1	2	37	0
I feel that attending the information clinic has given me better control of my situation	14	38	16	3	4	37	0
The information I received at the information clinic has increased my feeling of participation in the treatment	17	46	16	1	3	37	0
Would you recommend someone else with the same illness to attend the information clinic?	35	95	0		2	37	0
Do you think you had sufficient time to ask questions at the information clinic?	33	89	3		1	37	0
Did you find that you had the opportunity to ask questions about your thoughts regarding your treatment at your visit to the information clinic?	33	89	3		1	37	0
Did you find information missing at the information clinic?	3	8	26		7	36	1
At the information clinic, did you find that there was sufficient information about the scientific studies?	28	76	3		6	37	0
At the information clinic, did you find it difficult to decide if you want to participate in scientific studies?	5	14	32		0	37	0
Do you feel that communicating with the doctor at the information clinic has worked well?	33	89	4		0	37	0
Did you bring a family member to the information clinic?	24	65	13		0	37	0
If you brought a family member to the information clinic, do you feel that their attendance has increased the level of support you have received from family members?	21	88	1	11 NA	3	24	1
**At the information clinic several patients and family members attend at the same time...**	**Yes**		**No**		**Don't know**	**Total**	**Missing**
... did you find this a positive experience?	24	83	0		5	29	0
... did you feel that you could ask questions of a sensitive nature?	19	66	4		6	29	0
... did you feel it was a positive experience to meet others who are in a similar situation to you?	21	72	1		7	29	0
... did you miss not having an individual meeting with a doctor when you attended the information clinic?	3	10	20		6	29	0

## Discussion and Conclusions

### Discussion

In short, the patients attending the group consultation would recommend it to someone else and the group setting was a positive experience. A large proportion of patients agreed that the group consultation increased their sense of control and feeling of participation in their own treatment.

Patients planned for treatment for colorectal cancer attending the group consultation experienced that it increased their sense of control and their active participation in the treatment. This is corroborated by a previous study on the preoperative nurse information session as part of the enhanced recovery after surgery (ERAS) programme, which concluded that it made the patients more active in their own recovery after surgery [17]. The result of this study was that a large proportion of patients, who did not attend the group consultation, browsed the internet for more information on their cancer and the associated treatment. A previous study found that a majority of patients felt that the information on the internet made them feel empowered to make decisions about their health and helped them talk to their doctor about their health [18]. However, a number of the patients felt that the information could be overwhelming and made them aware of conflicting medical information about their cancer. This can be exemplified by a recent assessment of quality and accuracy of online information for patients with low anterior resection syndrome (LARS) where information on incidence often were lacking [19]. In contrast, the information given at the group consultation is based on science and proven experience from healthcare professionals. In conclusion, as a lot of patients with newly diagnosed colorectal cancer search the internet with varying results, the group consultation seems a good way of educating patients.

The strengths of this study were the study-specific questionnaire developed by interviews with patients and face validation before use. Further, the high response rate ensured reliable results. One limitation was the possible selection bias, meaning that all patients were invited to the group consultation but we found indications that those patients who chose to attend differed in some aspects, such as pain, anxiety, and distance to the hospital. Consequently, comparisons between groups were made with caution. Furthermore, the number of patients included did not provide power for statistical analyses regarding differences between the two groups of patients, but for this explorative study the design was considered suitable.

In a time of abundant information available from many sources, the validated information provided by healthcare personnel is important for patients recently diagnosed with colorectal cancer [20]. The patient browses different information sources, not always finding reliable or accurate descriptions of the disease and the treatment options. In light of this, one important task for the healthcare is to give patients facing cancer treatment accurate and reliable information. The consultation with the surgeon delivering the diagnosis of cancer can be stressful and retention of the large amounts of information difficult [21]. To educate patients through group consultations can be an appropriate method, to supplement the standard consultations where diagnosis is confirmed, and treatment options discussed. Some information before surgery could be given in a group setting with ample time for any questions the participating patients wish to raise. The questions raised by one patient may support others to actively participate and gather information.

### Conclusions

Preoperative group consultation is a feasible modality for delivering supplemental information to patients before treatment of colorectal cancer and to inform of ongoing clinical studies. The benefits of the group consultation following the standard visit to the outpatient clinic, could be to supplement the information found on the internet and through other sources, to answer and discuss issues appearing after the initial patient-surgeon consultation, and to increase of the patient’s participation in their treatment.

### Practical Implications

For the patients with newly diagnosed colorectal cancer who chose to attend the group consultation, it was found to have several benefits. To introduce it permanently in the care prior to surgery for colorectal cancer could probably increase the attending patients’ feeling of being in control and their active participation in their own treatment.

## Data Availability

The data is available through contact with the corresponding author.
